# A carbon cathode for lithium mediated electrochemical ammonia synthesis†

**DOI:** 10.1039/d4ee05669h

**Published:** 2025-03-20

**Authors:** Craig Burdis, Romain Tort, Anna Winiwarter, Johannes Rietbrock, Jesús Barrio, Maria Magdalena Titirici, Ifan E. L. Stephens

**Affiliations:** a Department of Materials, Royal School of Mines, Imperial College London London SW7 2AZ UK i.stephens@imperial.ac.uk; b Department of Chemical Engineering, Imperial College London London SW7 2AZ UK

## Abstract

To introduce the potential for tuneability of the cathode in lithium mediated ammonia synthesis, we report a carbon cathode which produces ammonia at a faradaic efficiency of 37%. This provides a basis to optimise properties of carbon electrodes to achieve high current densities and faradaic efficiencies.

## Abstract 2

To introduce the potential for tuneability of the cathode in lithium mediated ammonia synthesis, we report a carbon cathode which produces ammonia at a Faradaic efficiency of 37%.

## Graphical abstract

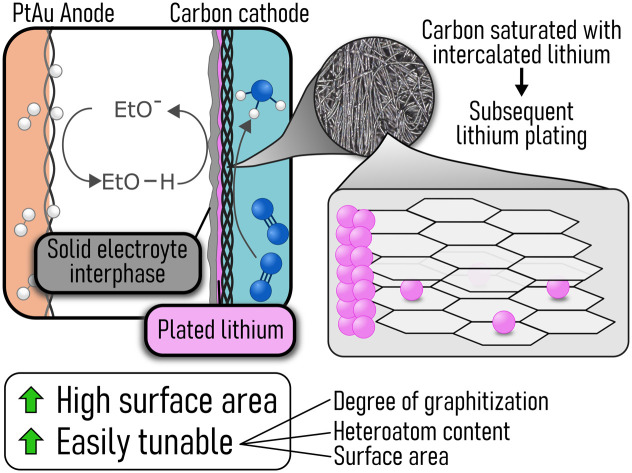

**Broader context**

Finding an alternative to the centralized and highly polluting Haber Bosch process for ammonia production could be a key step in decarbonization of the fertilizer industry; moreover, green ammonia has potential to be implemented as a carbon-free fuel. At the forefront of these efforts is the lithium mediated system, which has exhibited significant improvements in performance in terms of Faradaic efficiency and stability. There have however been a limited number of studies towards developing the cathode material, specifically efforts towards tuneable high surface area electrodes. Herein we employ a carbon gas diffusion layer as an alternative to stainless steel which paves the way towards these electrode goals. The Faradaic efficiency was improved from 17% ± 0.5% to 37% ± 4.5% by optimising the current density and ethanol concentration. This work opens the door to future optimisation of the carbon cathode to achieve high faradaic efficiency at higher, more industrially relevant current density.

## Introduction

Ammonia is a vital chemical that is primarily used to produce fertilizer for agriculture, but also has uses in the chemical industry as a commodity chemical.^1,2^ Globally, more than 180 million metric tons of ammonia are produced annually by the Haber–Bosch process^3^ which is responsible for around 1% of global carbon dioxide emissions and requires large, centralized facilities to be operated continuously. Developing an alternative process to produce ammonia with less environmental impact and in localizable devices would enable on-site and on-demand production that can be coupled to the ever-growing renewable energy sector. Alkali-metal mediated electrochemical processes, predominantly the lithium mediated system has proven to be the best candidate for activating dinitrogen (N_2_) since its revival in 2019.^4^ Initially developed by Tsuneto *et al.* in the 1990's,^5^ the system relies upon *in situ* electrodeposition of lithium metal from a lithium-based organic electrolyte, usually onto a metal working electrode.^5^ In conjunction with lithium metal, a solid electrolyte interphase forms which moderates the access of reagents to enable selective N_2_ reduction.^6,7^

Vast improvements in the Faradaic efficiencies of lithium mediated N_2_ reduction have resulted primarily from research focused on optimising the electrolyte^8–13^ or employing more industrially relevant cells such as a flow cell with gas diffusion electrodes.^12,14,15^ There have been a limited number of studies focused on the cathode material, most employ metal electrodes such as molybdenum^9,10^ or stainless steel.^12,14^ These electrodes exhibit low current density and electrochemical performance in terms of yield rate (nmol s^−1^ cm_geo_^−2^) towards ammonia due to their low surface area. In the state-of-the-art flow cell, the cathode is commonly stainless steel mesh which is also limited by its low surface area. A recent study by Li *et al.*^12^ achieved 60 mA cm_geo_^−2^ in a flow cell by employing a high surface area gas diffusion electrode which produced ammonia with a faradaic efficiency of 67%.^12^ The synthesis of such cathode materials, however, requires deposition of high surface area copper on to the stainless steel substrate. Copper is highly susceptible to corrosion in an ammonia rich environment, which may cause issues at high ammonia production rates.^16^

Carbon electrodes, both commercial and homemade, are commonplace in many electrochemical processes such as aqueous electrocatalysis^17–20^ and battery technologies.^21–24^ Carbon electrodes exhibit many favourable properties namely conductivity, high surface area, tunability and porosity.^25^ Several battery technologies employ carbon electrodes, most commonly lithium-ion batteries which store energy *via* intercalation of lithium ions into graphitic carbons.^23,26,27^ However, carbon has not been successfully employed in a flow cell for lithium mediated ammonia synthesis. Carbon's ability to intercalate lithium can be likened to metals alloying with lithium. The alloying of lithium with a metal is a descriptor established by Tsuneto *et al.*^5^ to screen electrode materials, who suggested that metals which alloy with lithium (aluminium and lead) would prevent lithium from activating N_2_.^5^ Translating this to carbon materials, the intercalation of lithium should prevent the reduction of N_2_ and production ammonia. In this assessment, the authors did not consider that the alloying energy is not constant with varying degree of lithiation.

In recent years, there has been increasing interest in lithium metal batteries which utilize lithium plating as an energy storage mechanism.^28^ For lithium-metal battery applications, Zhao *et al.* employed a commercial carbon gas diffusion layer as a host for dual-charge storage by lithium intercalation and plating.^28^ Plating occurred on the carbon electrode once the material was saturated with intercalated lithium.^28^ These results suggest the potential application of carbon materials as cathodes in the lithium mediated system, due to carbon's ability to plate lithium, although it is expected that there would be efficiency losses due to intercalation. An ideal carbon electrode for nitrogen reduction would behave similarly to one suited to lithium metal batteries, it should facilitate lithium plating. Carbon can also be used in batteries to protect copper electrodes from corrosion caused by electrolyte impurities such as hydrofluoric acid,^29,30^ meaning that carbon will be suitable for long-term nitrogen reduction.

**Fig. 1:**
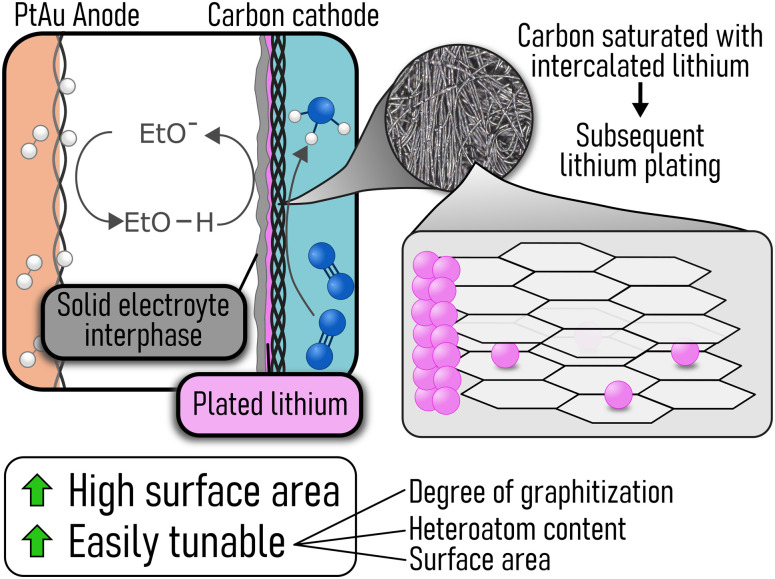
Schematic showing a flow cell for lithium mediated ammonia synthesis with a carbon cathode. The mechanism of lithium plating onto a carbon cathode is highlighted as well as the benefits of employing a carbon cathode.

Herein, we demonstrate that carbon can be used as a gas diffusion electrode for lithium mediated ammonia synthesis as a result of lithium plating occurring once the carbon is saturated with intercalated lithium (Fig. 1). We employed a commercial carbon gas diffusion layer (Freudenberg H15) as the cathode in a flow cell and assessed the performance across a range of current densities (−6 mA cm_geo_^−2^ to −60 mA cm_geo_^−2^) and ethanol concentrations (0.25 vol% to 1.0 vol%).

## Results

Freudenberg H15 was selected for this study due to the absence of a PTFE coating and microporous layer; we did not expect either of these features to be beneficial in a non-aqueous electrolyte. Freudenberg H15 was also found to have suitable physical properties for use in the flow cell, such as sufficient compressive strength and suitable flexibility. Other commercial carbon gas diffusion layers did not fulfil these physical requirements for the current flow cell design, see Text S2.1 (ESI†). Experiments, unless otherwise stated, were conducted in the electrolyte developed by Li *et al.*^12^ (1 M LiBF_4_ in diglyme with varying EtOH concentrations). A flow cell with a working area of 4 cm^2^ was employed with a Freudenberg H15 cathode, Pt wire pseudo reference electrode and PtAu anode (synthesized as reported by Fu *et al.*^14^). Fixed time current pulses were applied, for example: −6 mA cm_geo_^−2^ was applied for 30 seconds, then 0 mA cm_geo_^−2^ for 120 seconds. Increases in current density were matched with a reduction in the time of pulses and rests, applying the same multiplication factor (see Table S4.1 for pulsing conditions, ESI†).

**Fig. 2:**
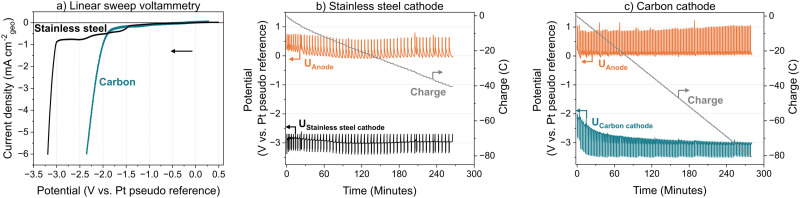
Electrochemistry data for carbon cathode in a flow cell: Cathode – Freudenberg H15, Pseudo-reference electrode – Pt wire, Anode – electrodeposited PtAu on stainless steel mesh; one-compartment flow cell, electrolyte flowing at 3 mL min^−1^, N_2_ flowing to the cathode at 30 mL min^−1^, H_2_ flowing to anode at 30 mL min^−1^; Potentials shown are not corrected for *iR* drop; 1 M LiBF4 in diglyme with 0.25 vol% EtOH. (a) Linear sweep voltammetry comparing stainless steel cathode and carbon cathode; 20 mV s^−1^ scan rate in negative direction from OCV. (b) Current pulsing with stainless steel mesh cathode; −6 mA cm_geo_^−2^ for 30 seconds then rest at 0 mA cm_geo_^−2^ until the potential reached −2.7 V *vs.* Pt. (c) Current pulsing with carbon cathode; −6 mA cm_geo_^−2^ for 30 seconds then rest at 0 mA cm_geo_^−2^ for 120 seconds.

Freudenberg H15 will herein be referred to as the carbon cathode, which exhibited notable differences in the electrochemical responses compared to the state-of-the-art stainless steel mesh (Fig. 2). During the linear sweep voltammetry, there is a significant current response at a potential positive of what is expected for lithium plating (around −3 V *vs.* Pt) (Fig. 2a). We have attributed this to lithium intercalation, which is comparable to battery literature.^31^ The potential response of the carbon cathode (Fig. 2c) during current pulsing differs from the response of the stainless steel mesh cathode (Fig. 2b) due to lithium intercalation. Whilst the potential of the stainless steel cathode remains constant around −3 V *vs.* Pt wire, the carbon cathode potential begins higher and then decreases as more charge is passed which correlates to more lithium intercalating.^21,28^ As intercalation proceeds, the lithium content in the carbon increases—resulting in a material more closely resembling lithium metal—thus shifting the potential towards that of lithium metal/plating. The potential of the carbon cathode then plateaus after about 60 minutes, suggesting full saturation of the carbon cathode with lithium; see S2.4 (ESI†) for discussion of this assignment. Subsequently, lithium is most likely plated onto the surface of the electrode, as the recorded potential suggests.^31–35^

Under the conditions established by Fu *et al.*^14^ (−6 mA cm_geo_^−2^ with 0.25 vol% EtOH), the carbon electrode produced ammonia at 17% ± 0.5% Faradaic efficiency. Argon blank experiments verified the source of the ammonia was from the input N_2_ gas. In an industrial setting, where devices would be operational for significantly longer periods of time, the efficiency losses associated with intercalation would become negligible. To corroborate this point and verify that the initial phase of the electrochemistry (where the potential of the carbon cathode decreases) does not contribute to the ammonia produced, we conducted a short-term experiment. The experiment was stopped before the potential plateaued, after 23 Coulombs had passed (Fig. S3.2, ESI†) and no ammonia was produced during this experiment. This supports the notion that the initial phase is due to intercalation, which does not contribute to ammonia production. As such, if we can neglect the charge passed during the initial charge (20 Coulombs) from the Faradaic efficiency calculation we can deduce that the Faradaic efficiency to NH_3_ in the following 20 coulombs would be as high as 24%.

**Fig. 3:**
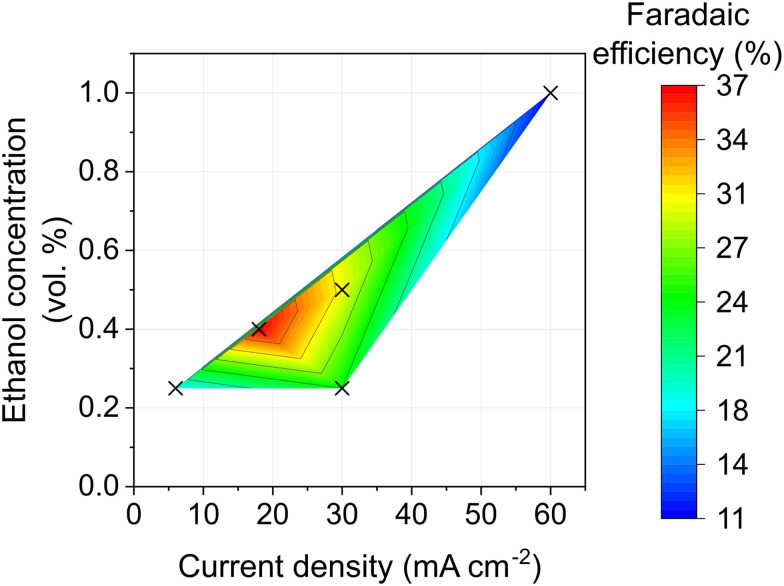
Contour plot showing the Faradaic efficiency (colour) of a carbon cathode in lithium mediated ammonia synthesis under varying current density (*x*-axis) and ethanol concentration (*y*-axis). Cathode – Freudenberg H15, pseudo-reference electrode – Pt wire, anode – electrodeposited PtAu on stainless steel mesh; one-compartment flow cell; electrolyte flowing at 3 mL min^−1^; N_2_ flowing to the cathode at 30 mL min^−1^; H_2_ flowing to anode at 30 mL min^−1^; 1 M LiBF_4_ in diglyme with varying EtOH concentration; varying current density pulsing.

Due to the carbon cathode having a higher surface area than the stainless steel mesh employed by Fu *et al.*,^14^ we expected that the optimal operating geometric current density would be higher for carbon. The complexity of the system and interplay between multiple parameters (current density, pulsing conditions and ethanol) results in a large space to be explored to optimise the Faradaic efficiency. We have conducted a preliminary screening of these conditions by operating at various current densities, pulse durations and ethanol concentrations. By varying these parameters, significant improvements in Faradaic efficiency were measured of up to 37% Faradaic efficiency at −18 mA cm_geo_^−2^ and 0.40 vol% EtOH (Fig. 3). These results highlight that further studies could engender significant improvements in Faradaic efficiency by optimising these operating conditions. However, at increased current densities with the PtAu anode employed, solvent oxidation occurs which will hinder the ammonia production.

The presence of water in lithium mediated ammonia synthesis has previously been shown to have a significant impact on the Faradaic efficiency.^10,12^ By drying the carbon cathode overnight (<40 mbar, 40 °C), a significant increase in Faradaic efficiency was measured; from 17% ± 0.5% to 37% ± 4.5% at −6 mA cm_geo_^−2^ with 0.25 vol% EtOH. An extended experiment (28 hours, 485C, S3.3, ESI†) under these conditions produced an average of 567 μmol ± 67 μmol (10.2 mg ± 1.03 mg, S4.5, ESI†) ammonia from two independent measurements with an average of 33.5% ± 3.5% Faradaic efficiency, negating the requirement for isotopically labelled experiments. Drying the carbon cathode also resulted in an increased proportion of ammonia produced in the gas phase, up to 50%. Understanding the cause for these preliminary findings will be the focus of future work.

## Outlook

Herein, we reported the first instance of a carbon electrode being used in a flow cell for lithium mediated ammonia synthesis at a maximum Faradaic efficiency of 37% ± 4.5% (−18 mA cm^−2^). We observed that the current density and associated reaction conditions (ethanol concentration and pulsing conditions) strongly influence the Faradaic efficiency. To operate at higher current density, the surface area of the anode must be increased which could be done by employing platinum on high surface area carbon powders.

We have provided the basis for developments in tuneable, high surface area electrodes to achieve high current densities and Faradaic efficiencies. Furthermore, an alternative gas diffusion electrode to stainless steel mesh has been presented which can itself be easily tuned for the reaction requirements or inspire research into other commercial or homemade carbon gas diffusion layers. There are several established deposition techniques for depositing catalysts onto carbon gas diffusion layers and we foresee that these methods would allow for increased current densities. Understanding the role of the structure and chemistry of this new class of cathode materials presents many opportunities for further research.

Future work should focus on tuning the properties of carbon electrodes, such as heteroatom content^36,37^ to increase the lithiophilicity and therefore propensity to plate lithium,^37^ or degree of graphitisation to reduce the energy losses caused by intercalation.^38^ Understanding the mechanism of suitable lithium deposition would lend itself to developing a structure–function relationship and therefore a more suitable carbon electrode. Carbon electrodes may exhibit higher Faradaic efficiencies in alternative chemistries, such as calcium.^39,40^ Therefore, beyond-lithium electrolytes as well as lithium-based electrolytes will be used to develop the ideal carbon electrode.

## Methods

Experiments were conducted in a flow cell with a working area of 4 cm^2^ and an electrolyte of 1 M LiBF_4_ in diglyme with varying ethanol concentrations (0.25–1.0 vol%). The electrodes were a Freudenberg H15 cathode, Pt wire pseudo reference electrode and PtAu anode (synthesized as reported by Fu *et al.*^14^ see S1.3 for experimental details, ESI†). The electrolyte was continuously flowed at 3 mL min^−1^, N_2_ flowed to the cathode at 30 mL min^−1^ and H_2_ flowed to the anode at 30 mL min^−1^. Current pulsing was employed, the standard pulses were 30 seconds at −6 mA cm_geo_^−2^, then 0 mA cm_geo_^−2^ for 120 seconds; changes in current density were matched with reduction in pulse parameters with the same multiplication factor. 80 Coulombs were passed during the current pulsing in all experiments. Detailed explanations of the experiments undertaken can be found in the ESI.†

## Author contributions

C. B., J. B., M.-M. T. and I. E. L. S. conceptualized the study. C. B. conducted the experiments and wrote the initial draft of the manuscript. J. R. took the SEM micrographs. C. B., R. T., A. W., J. B., M.-M. T. and I. E. L. S. contributed to scientific discussion and reviewed and edited the manuscript.

## Conflicts of interest

There are no conflicts to declare.

## Supplementary Material

EE-018-D4EE05669H-s001

## Data Availability

Data for this article, including data for figures and supplementary figures, are available at Imperial College London Research Data Repository at https://doi.org/10.14469/hpc/14875.
