# Supplementary material for: A carbon cathode for lithium mediated electrochemical ammonia synthesis
Source: Energy Environ Sci. 2025 Mar 20;18(12):5897–901. doi: 10.1039/d4ee05669h (PMC11948213; doi:10.1039/d4ee05669h)
Supplement: EE-018-D4EE05669H-s001 [file EE-018-D4EE05669H-s001.pdf]

Supplementary Information (SI) for Energy & Environmental Science.
This journal is © The Royal Society of Chemistry 2025

A carbon cathode for lithium mediated electrochemical ammonia synthesis

Craig Burdis,[a,b] Romain Tort,[b] Anna Winiwarter,[a] Johannes Rietbrock,[a] Jesús Barrio,[b] Maria Magdalena Titirici,[b] Ifan E. L. Stephens[a]*

[a] *Department of Materials, Royal School of Mines, Imperial College London SW7 2AZ, UK.*
[b] *Department of Chemical Engineering, Imperial College London, London SW7 2AZ, UK.*

Supplementary information

1. Materials and methods 3
 1.1. Materials 3
 1.2. Electrolyte preparation 3
 1.3. Electrode preparation 3
 1.4. Flow cell experiments 4
 1.5. Ion chromatography 4
 1.6. FE calculations 4
 1.7. Testing for contamination from the carbon electrode 4
 1.8. Scanning electron microscopy (SEM) of carbon electrodes before and after electrolysis 5
 1.9. Elemental analysis of carbon electrode 5
2. Supplementary text 6
 2.1. Commercial gas diffusion layers unsuitable for use in this flow cell design 6
 2.2. The negligible impact of water concentration variation when changing ethanol concentration 6
 2.3. Assessing contamination from the carbon electrode 6
 2.4. Corroborating lithium plating identification with calculation of charge required to fully intercalate carbon electrode 6
3. Supplementary figures 8
 3.1. One compartment flow cell employed 8
 3.2. Short nitrogen reduction experiment with carbon cathode 9
 3.3. Extended operation nitrogen reduction with carbon cathode 10
 3.4. Digital images of unsuitable carbon electrodes 11
 3.5. Bar chart of Faradaic efficiencies for carbon cathode with varying conditions 12
 3.6. Ion chromatography calibration 13
 3.7. Nitrite and nitrate calibration curve 14
 3.8. SEM micrographs of the carbon electrode before and after electrolysis 15
4. Supplementary tables 16
 4.1. Table of current pulsing conditions employed 16
 4.2. Table of nitrite contamination results 17

4.3. Table of nitrate contamination results 18
4.4. Table of ammonia contamination results 19
4.5. Table of water concentrations in electrolyte compositions and from carbon electrodes soaked in electrolyte overnight 20
4.6. Table of results from all nitrogen reduction experiments 21
4.7. Table of calculations for charge required to saturate carbon electrode at different lithium to carbon ratios 22
5. References 23

1. Materials and methods

1.1. Materials

Ammonium Standard for IC (Sigma Aldrich; NH_4^+: TraceCERT®), Chloroplatinic acid hexahydrate (Sigma Aldrich; $H_2PtCl_6 \cdot 6H_2O$: ACS reagent, ≥37.50% Pt basis), Freudenberg H15 was purchased from Fuel Cell Store, Gold(III) chloride trihydrate (Sigma Aldrich; $HAuCl_4 \cdot 3H_2O$: ACS reagent, ≥49.0 % Au basis), Hydrochloric acid (Sigma Aldrich; HCl: 30 %), Nitric acid (Sigma Aldrich; HNO_3: 70 %, ACS reagent), Nitrate Test (Sigma Aldrich, Supelco), Nitrite Test (Sigma Aldrich, Supelco) Platinum wire was purchased from Goodfellow inc., Potassium nitrate (Sigma Aldrich; KNO_3: ≥99.0 %), Potassium nitrite (Thermo Scientific; KNO_2, 97 %), Rubidium nitrate (Sigma Aldrich; $RbNO_3$: 99.7 % trace metal basis), Sodium carbonate (Sigma Aldrich; Na_2CO_3: anhydrous), Sodium hydrogen carbonate (Sigma Aldrich; $NaHCO_3$), Stainless steel mesh (Inoxia: 400 mesh), Stainless steel mesh (McMaster Carr: 325 × 2,300 mesh), Sulfuric acid (Sigma Adrich; H_2SO_4: 96 %, Suprapur®), ultrapure water (Sartorius, 18.2 MΩ resistivity).

The following chemicals were stored in an argon glovebox (<0.1 ppm H_2O, <0.1 ppm O_2, MBraun): Diglyme (Sigma Aldrich; 2-Methoxyethyl ether: anhydrous, 99.5 %), Ethanol (Acros Organics; EtOH: extra dry, absolute, 99.5 %), Tetrahydrofuran (Sigma Aldrich; THF: anhydrous, inhibitor-free, ≥99.9 %), Lithium tetrafluoroborate (Stanford Advanced Materials; $LiBF_4$: ≥99 %).

Ar (BOC, 99.9999 %), N_2 (BOC, 99.9999 %) and H_2 (BOC, 99.9995 %) were purified using NuPure purifiers which reduced contaminant levels of H_2O, H_2, CO_2, O_2, CO, nonmethane hydrocarbon (NMHC), CH_4, NH_3, NO_x to < 0.5 ppb.

1.2. Electrolyte preparation

$LiBF_4$ was dried for 72 hours at 120 °C under vacuum to remove residual water and other contaminants. Electrolytes were prepared in an argon-filled glovebox (MBraun, H_2O < 0.3 ppm, O_2 < 0.3 ppm).

1.3. Electrode preparation

Freudenberg H15 and stainless steel mesh (325 x 2300 mesh) were cut to size (2.25 cm x 2.25 cm) with a 4 cm^2 working area. To dry Freudenberg H15, the electrode was stored overnight in a vacuum oven at 40 °C and 0 mbar. The platinum wire pseudo reference was flame annealed prior to each experiment.

PtAu was electrodeposited onto stainless steel mesh as reported by Fu *et al.*[1]:

The PtAu electrode was prepared by hydrogen bubble templated electrodeposition onto stainless steel mesh. The stainless steel mesh was washed subsequently in water and ethanol, then sonicated in ethanol for 15 minutes and allowed to dry. The electrolyte for electrodeposition was 10 mM $H_2PtCl_6 \cdot 6H_2O$ and 10 mM $HAuCl_4 \cdot 3H2O$ in 3 M H_2SO_4. The electrochemical cell was a beaker with a Pt mesh counter electrode and stainless steel mesh working electrode. A current density of -0.2 A cm^{-2} was applied for 120 seconds. The stainless steel mesh electrode was removed and submerged in ultrapure water. The deposition was then repeated and the PtAu deposited stainless steel mesh was cleaned by repeatedly submerging in clean ultrapure water to remove electrolyte residue.

1.4. Flow cell experiments

The three electrode flow cell (working electrode area of 4 cm^2) was assembled with a PtAu anode (prepared as established by Fu *et al.*[1]), Pt wire pseudo reference and cathode (Freudenberg or stainless

steel mesh). The cell was purged with N_2 gas for 30 minutes before each experiment. Electrolyte lines were cleaned before each experiment by circulating THF for 10 minutes. Gas traps were prepared by filling 100 mL water into measuring cylinders (100 mL), with the N2 gas trap having HCl (250 µL, 4M) added to trap the ammonia as NH_4Cl. Gas and electrolyte lines were purged with N_2 then attached to the electrolyte reservoir. N_2 was flowed at 30 mL min^{-1} and electrolyte at 3 mL min^{-1}. The triple-phase boundary was established by altering the height of the gas exhausts in the water column, this was done until a continuous flow of electrolyte was visible and there was no flooding of the gas diffusion electrode. The anode gas was then switched to H_2 and the system was left to become saturated with H_2 (monitored by the open circuit voltage). The electrochemical workstation used was a BioLogic SP-150e. Linear sweep voltammetry was conducted at 20 mV s^{-1} in the negative direction until the current reached the operational current density. Current pulses of fixed time were then applied to the system, specific conditions can be found in Supplementary Table 4.1, until 80 C had passed. After electrochemistry, the system was left circulating for 30 minutes after which the electrolyte flow was reversed to empty the cell. Samples of the electrolyte and N_2 gas trap were taken for ion chromatography analysis. Samples were diluted in water such that concentrations measured were within the calibration curve. A Metrohm Eco IC was used for all measurements. The cell was disassembled and rinsed thoroughly with water then ethanol then left to dry.

1.5. Ion chromatography

A Metrohm Eco IC with autosampler and a Metrosep C 6 column was used for all measurements. The eluent was nitric acid (5.0 mM) and rubidium nitrate (0.34 µM). The regenerant was sodium carbonate (70 mM) and sodium hydrogen carbonate (70 mM). Samples were diluted between 10-100 times to ensure the concentration was within the calibration curve and to prolong the lifetime of the equipment and column.

1.6. FE calculations

$$FE_{NH_3} = \frac{3c_{NH_3}VF}{Q}$$

Where FE_{NH_3} is the Faradaic efficiency towards ammonia (%), F is the Faraday constant (96485 C mol^{-1}), c_{NH_3} is the concentration of ammonia measured from ion chromatography (mol dm^{-3}), V is the volume of electrolyte (dm^3) and Q is the charge passed during the experiment (C).

1.7. Testing for contamination from the carbon electrode

Freudenberg H15 (1 cm^2) was soaked in electrolyte (1 M $LiBF_4$ in diglyme with 0.25 vol.% EtOH, 1 mL) and separately in water overnight. 200 µL was sampled and diluted up to 2 mL with water. Nitrite test (Sigma Aldrich, 25 mg) was added and left for 15 minutes for the reaction to occur. Samples were then analysed using UV-Vis from 900 nm to 200 nm and the peak absorbance at 550 nm was used to calculate the NO_2^- concentration using a calibration curve. This procedure was repeated using a Nitrate test (Sigma Aldrich), taking a maximum absorbance at 340 nm.

Freudenberg H15 (1 cm^2) was soaked in electrolyte (1 M $LiBF_4$ in diglyme with 0.25 vol.% EtOH, 1 mL) and separately in water overnight. 500 µL was sampled and diluted up to 5 mL with water. Samples were then tested for ammonia using ion chromatography.

1.8. Scanning electron microscopy (SEM) of carbon electrodes before and after electrolysis

Pristine Freudenberg H15 and post-extended experiment Freudenberg H15 (air-exposed) was cut using scissors. A piece of the post-electrolysis sample was washed in water to remove the solid electrolyte interphase then all samples were dried in glass vials on a hot plate set to 50 °C. The samples were fixed to an SEM sample holder using carbon tape. All micrographs were taken using a Gemini 1 Zeiss Sigma 300 microscope.

1.9. Elemental analysis of carbon electrode

Pristine Freudenberg H15 and post-argon blank experiment Freudenberg H15 (air-exposed) was cut using scissors. The post-argon blank experiment carbon was washed in water and sonicated for 15 minutes in water to remove residual solid electrolyte interphase and subsequently dried. The electrodes were weighed in a tin capsule then transferred to a Flash 2000, Organic Elemental Analyzer, Thermo Scientific for elemental CHN analysis.

2. Supplementary text

2.1. Commercial gas diffusion layers unsuitable for use in this flow cell design

Other commercial gas diffusion layers that were tested and found to be unsuitable for use in this flow cell design were Plain Carbon Cloth (purchased from Fuel Cell Store), Toray Carbon Paper (purchased from Fuel Cell Store) and Sigracet 39AA (purchased from Fuel Cell Store). A triple phase boundary could not be established with the Plain Carbon Cloth and due to its flexibility, the cell shorted as the carbon deformed with the gas flow. Toray Carbon Paper was too fragile for use in this flow cell as tightening the cell with the minimum force required to prevent the cell leaking resulted in the carbon paper being destroyed (Supplementary Figure 4a). Sigracet 39AA was destroyed at the point where the gasket seals the cell which produced carbon debris; this debris blocked the flow of electrolyte in the cell and prevented a triple phase boundary from being established (Supplementary Figure 4b).

2.2. The negligible impact of water concentration variation when changing ethanol concentration

As it is known that water concentration has a strong impact on the performance of lithium mediated ammonia synthesis, we conducted measured the concentration of water in the electrolytes employed in this study. It was found that varying ethanol concentration did not have a significant impact on water concentration, Supplementary Table 4.5.

2.3. Assessing contamination from the carbon electrode

To validate the veracity of our results, several contamination control experiments were conducted. In this work we were employing a previously validated experimental setup, such as gas purity, gas contaminants and electrolyte contaminants. The only unverified component of this work was the carbon electrode, as such we assessed the NO_2^-, NO_3^- and NH_4^+ contribution from the pristine material, Supplementary Information 1.7. There was not found to be any NO_2^-, NO_3^- or NH_4^+ contribution from the material, beyond that found in blank water, Supplementary Tables 4.2, 4.3 and 4.4 respectively.

In line with the recommendations of Iriawan *et al.*[2], we have fulfilled the criteria of $mass_{prod} > 2mass_{sys}$ and $C_{prod} > 100ppm$ to not require isotopically labelled experiments. Elemental analysis (CHN) of the pristine carbon electrode detected 3.94 wt. % ± 0.04 wt. % of the electrode to be nitrogen. After an argon blank experiment, the carbon cathode was found to have 3.80 wt. % ± 0.05 wt. % indicating that there was no significant decrease in the nitrogen content of the carbon. In reference to the recommendations of Iriawan *et al.*[2], the mass of nitrogen in the system would equate to 0.8 mg in a 4 cm^2 electrode. After passing 80 C of charge, 1.8 mg ± 0.24 mg ammonia was produced. After 480 C of charge was passed, 10.8 mg ± 0.47 mg ammonia was produced.

2.4. Corroborating lithium plating identification with calculation of charge required to fully intercalate carbon electrode

To corroborate attributing the plateaued potential to lithium plating, we calculated the amount of charge required to saturate the carbon electrode with lithium. We calculated across a range of assumed ratios of lithium to carbon: LiC_{18}, LiC_{12} and LiC_6, which are expected ratios in line with the works of Zhao *et al.*[3] in which a similar gas diffusion electrode was employed. It was found that the charge required to saturate the carbon electrode with a ratio of LiC_{18} was 16 C, LiC_{12} was 24 C and for LiC_6 was 48 C, Supplementary Table 4.7. This suggests that the plateauing potential does correspond to the carbon being saturated with lithium; however, we cannot conclusively assign the ratio of lithium to carbon, nor

the exact plateauing potential with electrochemical data alone. Techniques such as *in situ* Raman would provide such insight.[4]

3. Supplementary figures

3.1. One compartment flow cell employed

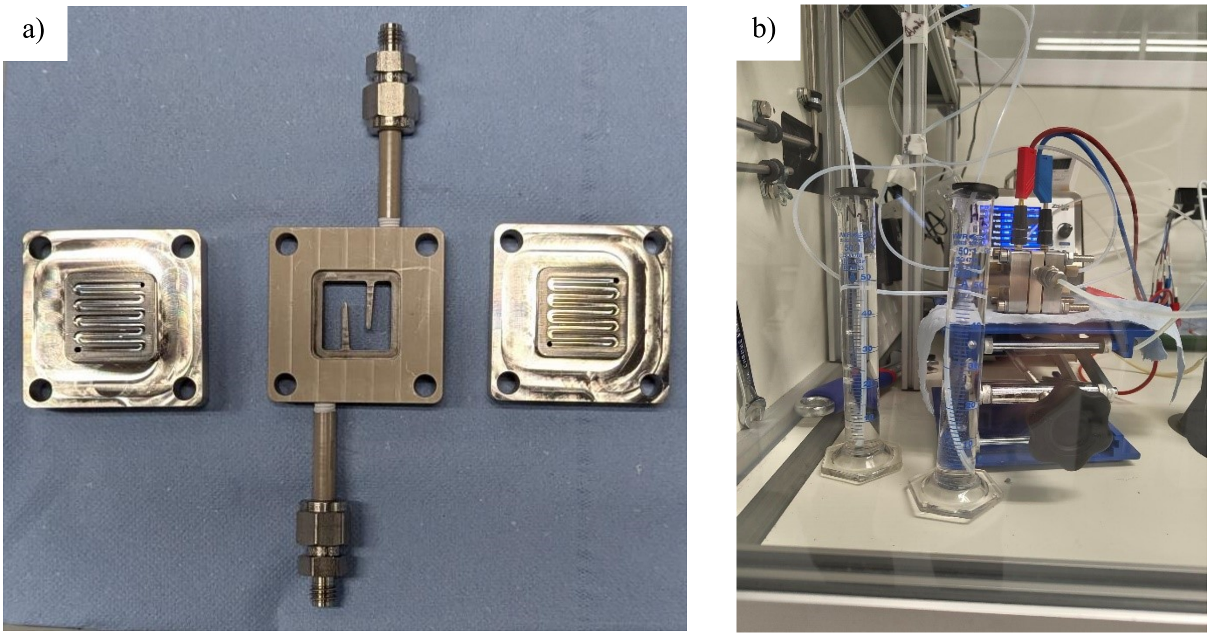

Supplementary Figure 1. Digital images of the one compartment flow cell used for electrochemical measeurements. 4 cm^2 active electrode area, with 4 mm separation between cathode and anode. a) Disassembled cell made up of two stainless steel gas flow fields and one PEEK electrolyte flow field (rotated for image, all flow fields are horizontal when assembled). b) Assembled flow cell in operation.

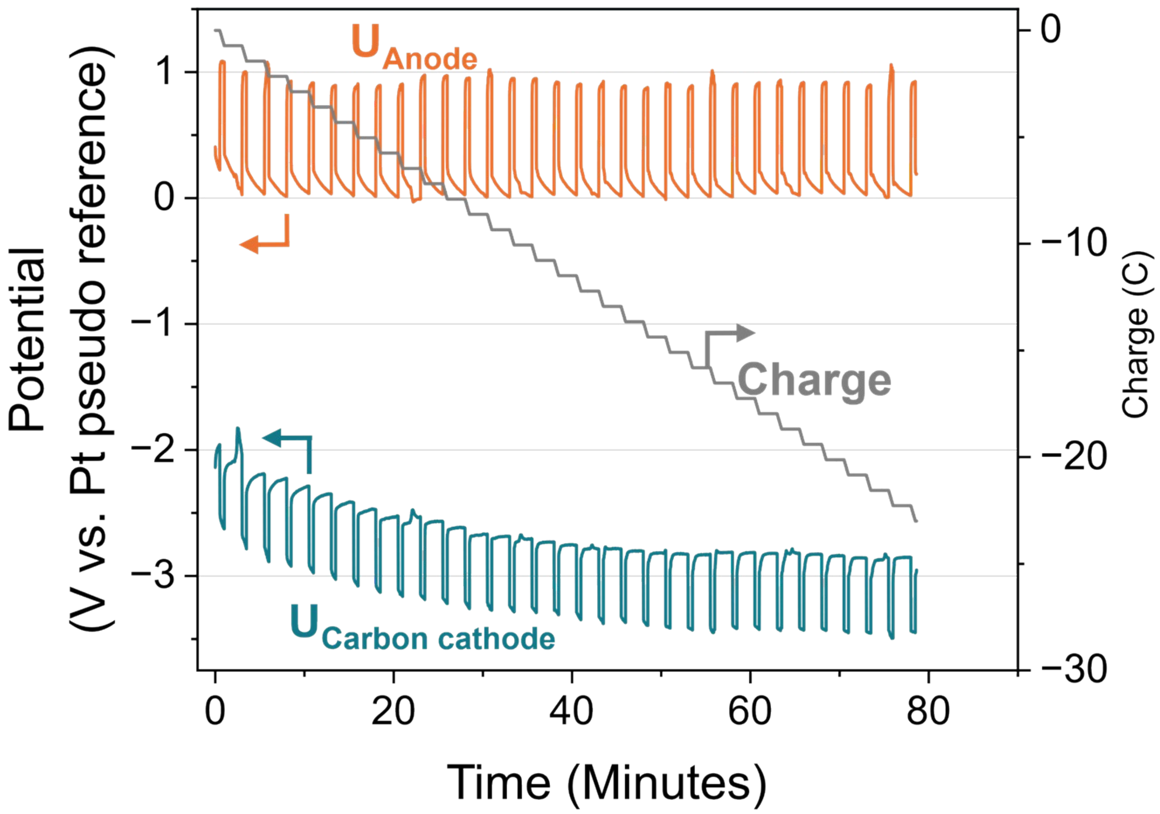

Supplementary Figure 2. Potential response from current pulsing of a carbon cathode in a flow cell, short term experiment: Cathode – Freudenberg H15, pseudo-reference electrode – Pt wire, anode – electrodeposited PtAu on stainless steel mesh; One-compartment flow cell, electrolyte flowing at 3 mL min^{-1}, N_2 flowing to the cathode at 30 mL min^{-1}, H_2 flowing to anode at 30 mL min^{-1}; Potentials shown are not corrected for iR drop; 1 M $LiBF_4$ in diglyme with 0.25 vol. % EtOH; current pulsing conditions: -6 mA cm$^{-2}_{geo}$ for 30 seconds then rest at 0 mA cm$^{-2}_{geo}$ for 120 seconds.

3.2. Short nitrogen reduction experiment with carbon cathode

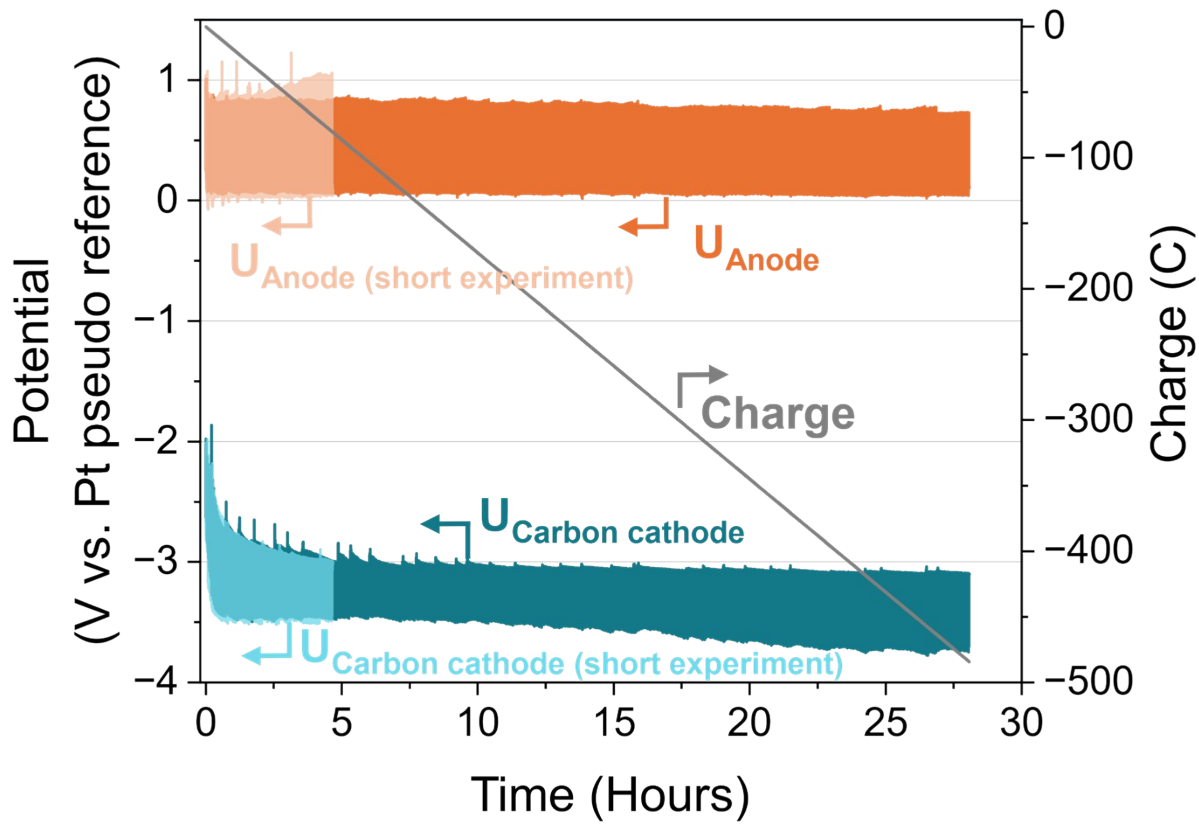

3.3. <u>Extended operation nitrogen reduction with carbon cathode</u>

Supplementary Figure 3. Potential response from current pulsing of a carbon cathode in a flow cell, extended experiment with short experiment overlayed in lighter colours: Cathode – Freudenberg H15, pseudo-reference electrode – Pt wire, anode – electrodeposited PtAu on stainless steel mesh; One-compartment flow cell, electrolyte flowing at 1 mL min^{-1}, N_2 flowing to the cathode at 30 mL min^{-1}, H_2 flowing to anode at 30 mL min^{-1}; Potentials shown are not corrected for iR drop; 1 M $LiBF_4$ in diglyme with 0.25 vol. % EtOH; current pulsing conditions: -6 mA cm$^{-2}_{geo}$ for 30 seconds then rest at 0 mA cm$^{-2}_{geo}$ for 120 seconds.

3.4. <u>Digital images of unsuitable carbon electrodes</u>

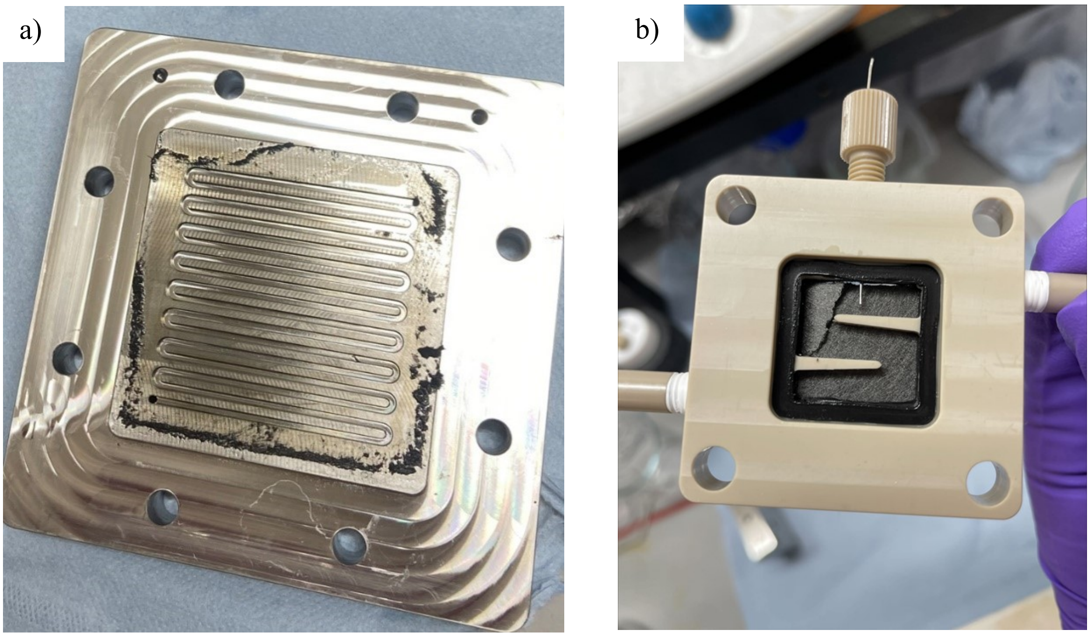

Supplementary Figure 4. Digital images of carbon electrodes unsuitable for use in this flow cell design: a) residual carbon from Sigracet 39AA produced from clamping cell to seal. b) Toray Carbon Paper cracked as a result of clamping cell to seal.

3.5. Bar chart of Faradaic efficiencies for carbon cathode with varying conditions

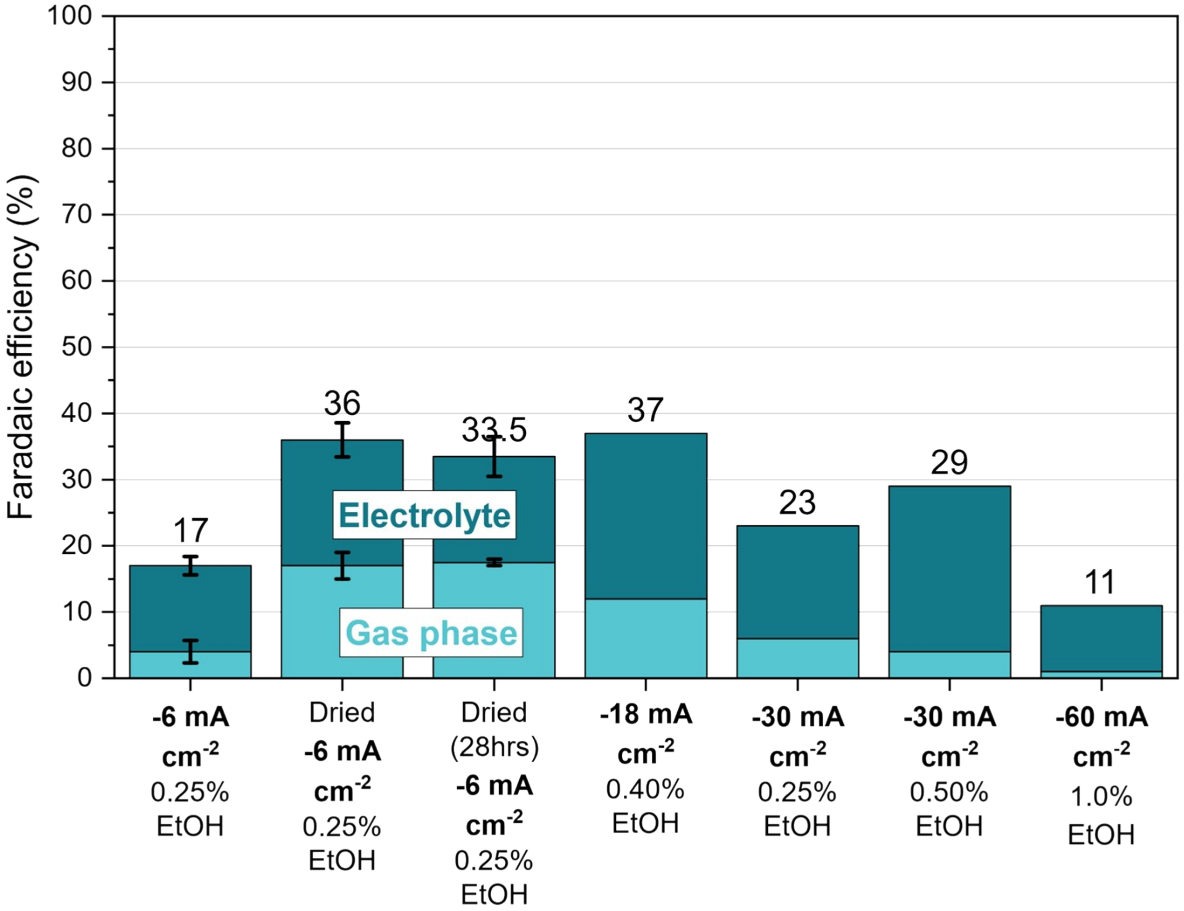

Supplementary Figure 5. Bar chart of Faradaic efficiencies of carbon cathode with varying current densities, ethanol concentrations and material drying: Cathode – Freudenberg H15, pseudo-reference electrode – Pt wire, anode – electrodeposited PtAu on stainless steel mesh; One-compartment flow cell, electrolyte flowing at 3 mL min-1, N_2 flowing to the cathode at 30 mL min^{-1}, H_2 flowing to anode at 30 mL min^{-1}; Potentials shown are not corrected for iR drop; 1 M $LiBF_4$ in diglyme with 0.25 vol. % EtOH.

3.6. Ion chromatography calibration

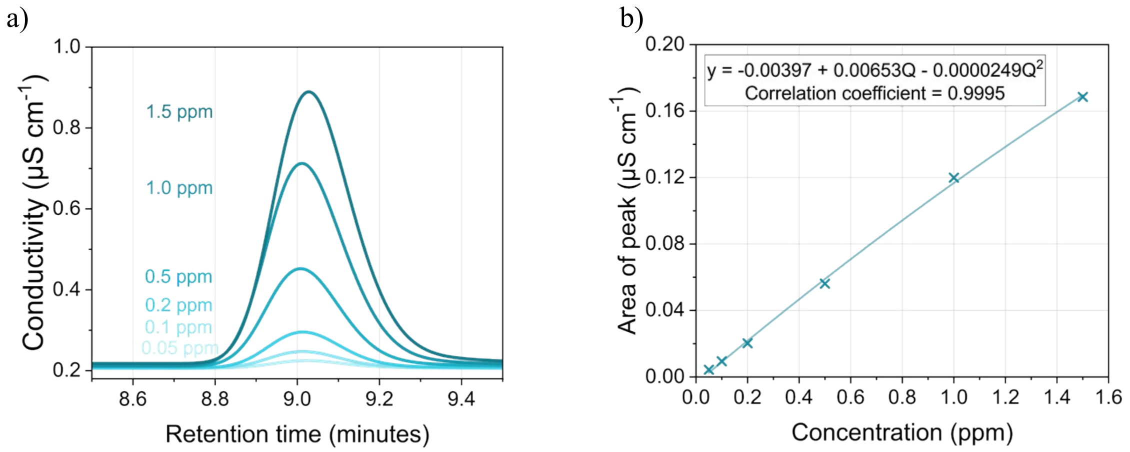

Supplementary Figure 6. Replotted data taken from ion chromatography software used for calibration: a) Ammonium peak in chromatograph for given concentrations of standard solutions. b) Calibration curve of ammonium concentration (x-axis) vs area of the ammonium peak (y-axis).

3.7. Nitrite and nitrate calibration curve

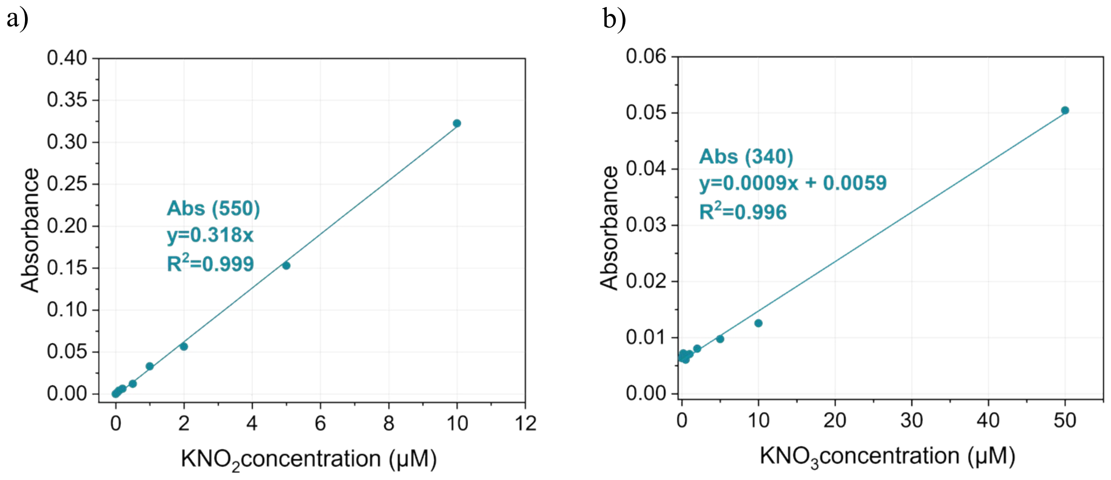

Supplementary Figure 7. Calibration curves for a) nitrite concentration and b) nitrate concentration.

3.8. SEM micrographs of the carbon electrode before and after electrolysis

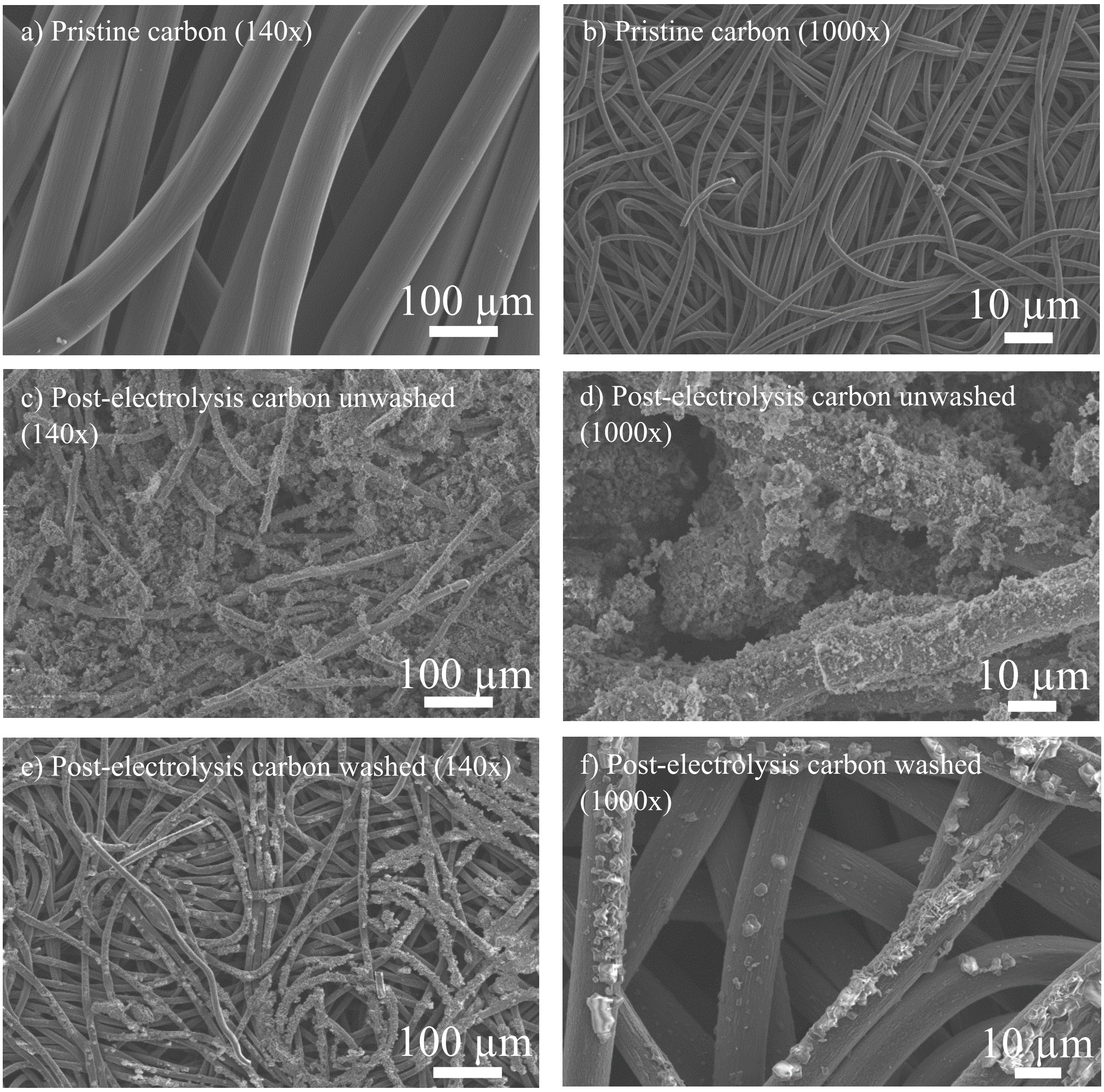

Supplementary Figure 8. SEM micrographs of carbon electrodes before and after electrolysis. All micrographs were taken using a Gemini 1 Zeiss Sigma 300 SEM with an accelerating voltage of 5000 V: a-b) Pristine carbon cathode before experiment, c-d) Carbon cathode after extended operation nitrogen reduction unwashed and air-exposed, e-f) Carbon cathode after extended operation nitrogen reduction washed in water and air-exposed.

4. Supplementary tables

4.1. Table of current pulsing conditions employed

Current density (mA cm^{-2}_{geo})	Current pulse duration (s)	Rest duration at 0 mA cm^{-2}_{geo} (s)
-6	30	120
-18	10	40
-30	6	20
-60	1	10

4.2. Table of nitrite contamination results

**Sample**	**Wavelength (nm)**	**Max A**	**Concentration (µM)**	**Amount of nitrite (µmol)**
Blank water	550	0.017	0.541	0.0011
Carbon soaked in water (1)	550	0.014	0.454	0.0009
Carbon soaked in water (2)	550	0.014	0.446	0.0009
Water + NO2 (5 µM)	550	0.203	6.375	0.0128
Electrolyte blank	550	0.012	0.383	0.0008
Carbon soaked in electrolyte (1)	550	0.009	0.298	0.0006
Carbon soaked in electrolyte (2)	550	0.021	0.647	0.0013
Electrolyte + NO2 (5 µM)	550	0.185	5.803	0.0116

4.3. <u>Table of nitrate contamination results</u>

**Sample**	**Wavelength (nm)**	**Max A**	**Concentration (μM)**	**Amount of nitrite (μmol)**
Carbon soaked in water (1)	340	0.0050	-1.0508	N/A
Carbon soaked in water (2)	340	0.0048	-1.2543	N/A
Carbon soaked in electrolyte (1)	340	-0.0003	-6.8689	N/A
Carbon soaked in electrolyte (2)	340	0.0003	-6.1803	N/A

4.4. Table of ammonia contamination results

Sample	Ammonia concentration (ppm)	Amount of ammonia (µmol)
Blank water	0.012	0.0001
Carbon soaked in water (1)	0.017	0.0001
Carbon soaked in water (2)	0.015	0.0001
Carbon soaked in electrolyte	Not detectable	Not detectable

4.5. Table of water concentrations in electrolyte compositions and from carbon electrodes soaked in electrolyte overnight

Sample	Water concentration (ppm) ± standard deviation (two measurements)
1 M $LiBF_4$ in diglyme	158
1 M $LiBF_4$ in diglyme + 0.25 vol.% EtOH	160 ± 14
1 M $LiBF_4$ in diglyme + 1.0 vol.% EtOH	159 ± 0.5
Dried carbon overnight in electrolyte	153 ± 0.0
Wet carbon overnight in electrolyte	180 ± 10.5

4.6. Table of results from all nitrogen reduction experiments

Cathode gas	Electrode dried	Current density (mA cm^{-2}_{geo})	Ethanol concentration (vol. %)	Charge passed (C)	Ammonia produced (μmol)
Ar	Not dried	6.0	0.25	80.8	0.55
N_2	Not dried	6.0	0.25	80.5	48.30
N_2	Not dried	6.0	0.25	80.0	49.13
N_2	Not dried	6.0	0.25	80.0	47.92
N_2	40 °C, 0 mbar	6.0	0.25	80.0	89.24
N_2	40 °C, 0 mbar	6.0	0.25	80.0	97.32
N_2	40 °C, 0 mbar	6.0	0.25	80.8	120.32
N_2	40 °C, < 40 mbar	6.0	0.25	484	623.84
N_2	40 °C, < 40 mbar	6.0	0.25	485	572.20
N_2	Not dried	18.0	0.40	84.1	106.2
N_2	Not dried	30.0	0.25	82.7	71.00
N_2	Not dried	30.0	0.50	81.7	81.98
N_2	Not dried	60.0	1.00	80.0	29.76

4.7. Table of calculations for charge required to saturate carbon electrode at different lithium to carbon ratios

Mass of electrode (mg)	40		
Carbon content (wt. % from CHN)	89		
Mass of carbon (mg)	35.6		
Moles of carbon (mmol)	3.0		
Assumed Li:C ratio	**LiC_6**	**LiC_{12}**	**LiC_{18}**
Moles of Li (mol)	0.0005	0.0002	0.0002
Faraday constant (C/mol)	96485	96485	96485
Charge for that amount of Li (C)	48	24	16

5. References

1 X. Fu, J. B. Pedersen, Y. Zhou, M. Saccoccio, S. Li, R. Sažinas, K. Li, S. Z. Andersen, A. Xu, N. H. Deissler, J. Bjarke, V. Mygind, C. Wei, J. Kibsgaard, P. C. K. Vesborg, J. K. Nørskov and I. Chorkendorff, *Science (1979)*, 2023, **379**, 707–712.

2 H. Iriawan, S. Z. Andersen, X. Zhang, B. M. Comer, J. Barrio, P. Chen, A. J. Medford, I. E. L. Stephens, I. Chorkendorff and Y. Shao-Horn, *Springer Nature*, 2021, preprint, DOI: 10.1038/s43586-021-00053-y.

3 T. Zijian Zhao, W. M. Dose and M. F. L. De Volder, *Batter Supercaps*, 2022, **5**, 1–8.

4 H. Yadegari, M. A. Koronfel, K. Wang, D. B. Thornton, I. E. L. Stephens, C. Molteni, P. D. Haynes and M. P. Ryan, *ACS Energy Lett*, 2021, **6**, 1633–1638.
